# Relation of fibroblast growth factor receptor 2 expression to hepatocellular carcinoma recurrence after liver resection

**DOI:** 10.1371/journal.pone.0227440

**Published:** 2020-01-15

**Authors:** Baek Gyu Jun, Woong Cheul Lee, Jae Young Jang, Soung Won Jeong, Young Chang, Sae Hwan Lee, Young don Kim, Sang Gyune Kim, Gab Jin Cheon, Young Seok Kim, Hong Soo Kim, So Young Jin

**Affiliations:** 1 Department of Internal Medicine, University of Ulsan College of Medicine, Gangneung Asan Hospital, Gangneung, South Korea; 2 Institute for Digestive Research, Digestive Disease Center, Department of Internal Medicine, College of Medicine, Soonchunhyang University, Seoul, Korea; 3 Department of Internal Medicine, College of Medicine, Soonchunhyang University, Cheonan, Korea; 4 Department of Internal Medicine, College of Medicine, Soonchunhyang University, Bucheon, Korea; 5 Department of Pathology, College of Medicine, Soonchunhyang University, Seoul, Korea; University of Navarra School of Medicine and Center for Applied Medical Research (CIMA), SPAIN

## Abstract

**Background:**

Hepatocellular carcinoma (HCC) recurrence after liver resection depends upon the stage and histological grade of the tumor and the expression of certain biomarkers. However, it remains unclear which of these factors has the highest predictive value regarding HCC recurrence after surgical resection.

**Methods:**

This study investigated the associations among clinicopathological characteristics, expression of biomarkers, and HCC recurrence after liver resection. Fifty-four patients having undergone liver resection for HCC were enrolled prospectively, and their data were analyzed retrospectively. Evaluated variables were clinical data, laboratory findings, modified Union for International Cancer Control (UICC) stage, vascular invasion, histological differentiation, and immunohistochemical staining for fibroblast growth factor receptor 2 (FGFR2), vascular endothelial growth factor, and tumor-necrosis-factor-related apoptosis-inducing ligand receptors 1 and 2.

**Results:**

Mean patient age was 58.6 years (range, 30–71), and the mean and SD for follow-up duration were 51.2 ± 34.8 months. Cumulative 1-, 3-, and 5-year recurrence rates were 32.9%, 53.6%, and 68.1%, respectively. In univariate analysis, FGFR2 (*p* = 0.026) and Edmonson-Steiner grade (E-S grade) (*p* = 0.030) were associated with recurrence after resection in HCC patients. In multivariate analyses, increased FGFR2 expression (*p* = 0.017) was the only significant predictor of HCC recurrence.

**Conclusions:**

High FGFR2 expression had marginal association with poor E-S grade (*p* = 0.056). More intensive surveillance of HCC recurrence is warranted in HCC patients with increased FGFR2 expression.

## Introduction

Hepatocellular carcinoma (HCC), a common type of primary liver cancer, is malignant with the fifth highest incidence and the third highest mortality rates worldwide. HCC accounts for nearly 700,000 deaths per year, and the incidence of HCC continues to increase [1, 2]. Approximately 30% of newly diagnosed patients are eligible for potentially curative therapies, such as liver transplantation, hepatic resection, or percutaneous ablation [3], with hepatic resection the predominant treatment modality [4]. Ideal candidates are patients with single nodules, well-preserved liver function, absence of portal hypertension, and no extrahepatic spread [5]. Unfortunately, long-term survival remains far from satisfactory due to the extremely high incidence of postoperative recurrence, with reported 5-year cumulative recurrence rates from 77 to 100%; of these, 80 to 95% occur in the remaining liver [6–9].

HCC recurrence after liver resection depends upon the stage and histological grade of the tumor and the expression of certain biomarkers [10]. Effective prediction of recurrence and management of recurrent tumors are important for improving overall survival (OS) after surgical resection. However, it remains unclear which of these factors has the greatest predictive value for HCC recurrence after surgical resection.

Expression analysis of several biomarkers by immunohistochemical (IHC) staining of tumor specimens might help predict HCC prognosis after liver resection and liver transplantation [11]. Mocchetti et al. found that fibroblast growth factor receptor (FGFR) 2 was frequently expressed in hepatoma-derived cell lines while healthy human primary hepatocytes did not express FGFR2; his group proposed that HCC proliferation might be regulated through autocrine or paracrine mechanisms mediated by FGF/FGFR2 [12].

FGFR is reportedly involved in the progression of many cancers [13–16]. Increased FGFR2 expression in HCC has been correlated with decreased tumor differentiation [17]. VEGF is an important mediator of tumor angiogenesis, and high serum VEGF levels have been shown to predict poor survival in several cancers [18]. Induction of apoptosis through the interaction of TRAIL with its receptors on the surface of cancer cells is a well-described mechanism of tumor surveillance [19], and the in vivo importance of loss of sensitivity to TRAIL-mediated apoptosis has been demonstrated by multiple clinical studies showing a correlation between TRAIL receptor expression, poor prognosis, and tumor recurrence [20].

The present study investigated the clinicopathological characteristics and expression of biomarkers of risk factors of HCC recurrence after liver resection, including FGFR2, VEGF, TRAIL-R1, and TRAIL-R2, in patients with HCC.

## Material and methods

### Patients

Patient data in the hepatic surgery database at the Soonchunhyang University Hospital (Seoul, South Korea) was prospectively collected for this retrospective study. Institutional review board approval was obtained from the Soonchunhyang university prior to data analyses (No. 2012–173). Written informed consent was obtained from all patients. Sixty-six patients underwent hepatectomy for HCC between June 2003 and November 2012 at Soonchunhyang University Hospital. All of these patients were Barcelona-Clinic Liver Cancer (BCLC) stage 0 or A [21, 22]. Curative resection was defined as the removal of all recognizable tumors with clean margins; a resection tumor margin of 0 mm was defined as curative as long as no tumor invasion was observed at the surgical cut surface [23]. Retrospective examination of these data led to the exclusion of 12 patients due to combined hepatocellular-cholangiocarcinoma (*n* = 4), death in hospital due to postoperative hepatic failure (*n* = 1), or noncurative resection (*n* = 7). A modified Union for International Cancer Control (UICC) staging classification (I–III) [24] was used to determine the clinical stage of each tumor.

### Follow-up parameters

After resection, follow-up analyses of alpha fetoprotein (AFP) and imaging were conducted. Imaging was performed using computed tomography (CT) or magnetic resonance imaging (MRI). Screening for AFP and imaging were conducted one month after resection, then every 3 months thereafter for one year, and then every 6 months thereafter through the end of the study period in April 2016. Recurrence was diagnosed based on the combined findings of these clinical examinations. All follow-up data were summarized as of April 2016.

### IHC staining of biomarkers

Tumor samples used in this study for the determination of FGFR2, VEGF, TRAIL-R1, and TRAIL-R2 were obtained from resected liver cancer specimens. Representative paraffin-embedded tissue samples were serially cut and mounted on glass slides for immunohistochemistry. The avidin-biotin-peroxidase complex technique was used with a Benchmark XT Autoimmunostainer (Ventana Medical Systems/Roche, Tucson, AZ, USA) for the IHC process. Each section was treated using the UltraView Universal diaminobenzidine detection kit (Ventana Medical Systems/Roche). Antigen retrievals were performed with enzyme method using protease of Ventana DAB Universal Kit (Ventana Medical Systems/Roche). The following primary antibodies were applied:

- FGFR2 (ab10648; Abcam, Cambridge, UK). After pretreatment with protease for 16 min, sections were incubated with antibody against FGFR2 at a dilution of 1:1,000 for 48 min at room temperature (RT).
- VEGF (A-20, sc-152; Santa Cruz Biotechnology, Santa Cruz, CA, USA). After antigen-retrieval pretreatment for 60 min, sections were incubated with antibody against VEGF at a dilution of 1:100 for 48 min at RT.
- TRAIL-R1 (ab8414; Abcam) After antigen-retrieval pretreatment for 30 min, sections were incubated with antibody against TRAIL-R1 at a dilution of 1:400 for 32 min at RT.
- TRAIL-R2 (ab8416; Abcam) After antigen-retrieval pretreatment for 60 min, sections were incubated with antibody against TRAIL-R2 at a dilution of 1:200 for 32 minutes at 37°C.

Lymphoid cells were used as positive controls for TRAIL-R1 and TRAIL-R 2 to optimize primary antibody titers. Bile duct tissue was used as an internal control for FGFR2 expression.

### Immunoreactive scoring

**Fig 1:**
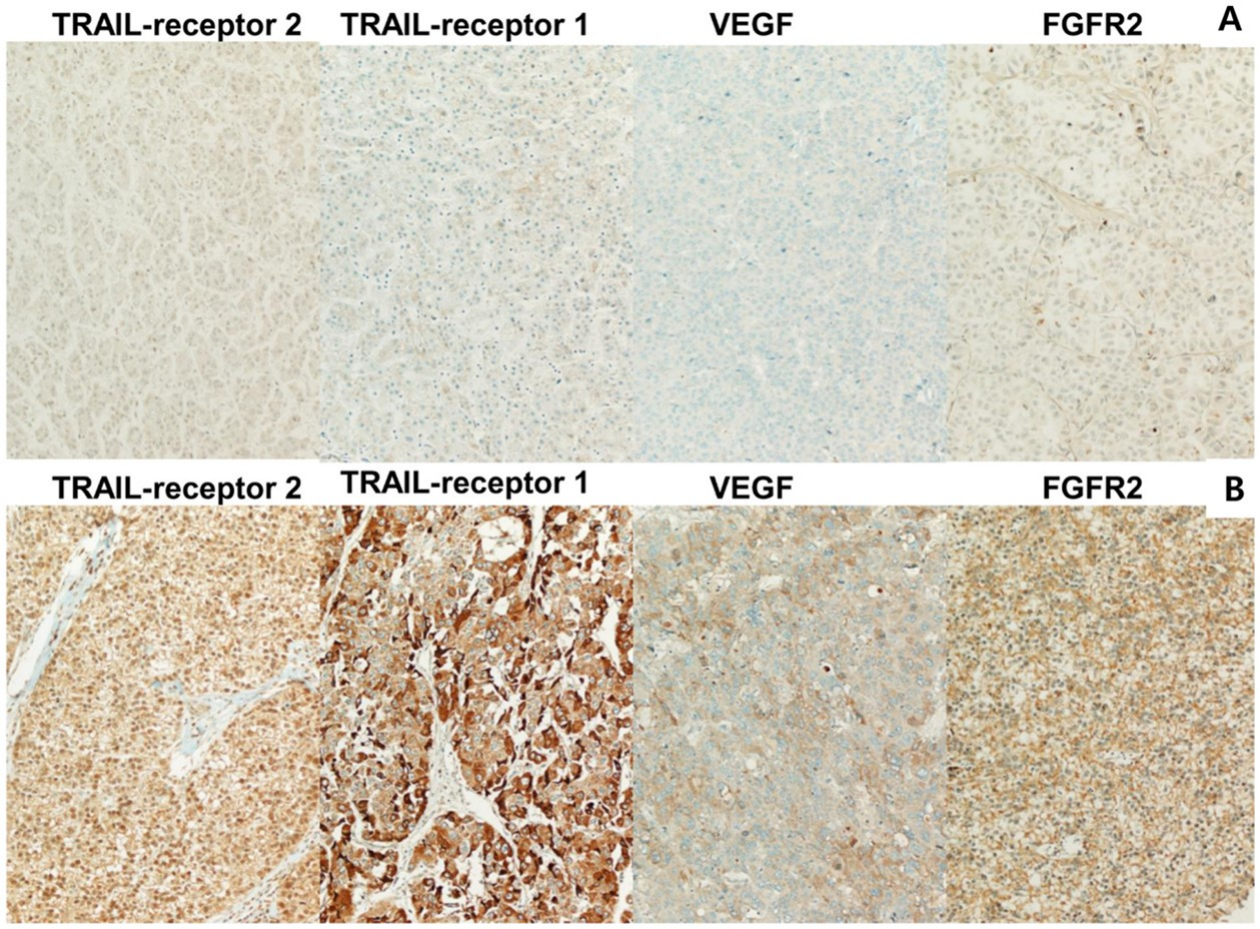
IHC stain IHC staining for FGFR2, VEGF, TRAIL receptor 1, and TRAIL receptor 2. (A) IRS grade 0 showing scanty dark brown granular cytoplasmic and/or membranous staining in neoplastic hepatocytes (200X), and (B) IRS grade 3 showing strong dark brown stain in neoplastic hepatocytes (200X).

Dark brown granular cytoplasmic and/or membranous staining in neoplastic hepatocytes was considered a positive result. Grading was achieved according to the multiplication of staining intensity by percentage of positive cells. The grades were as follows: grade 0, negative and trace; grade 1, weak; grade 2, medium; and grade 3, strong. The percentage of positive cells was graded as follows: grade 0, < 5%; grade 1, 5–29%; grade 3, 30–49%; and grade 4, > 50%. The multiplied immunoreactive score (IRS) was graded as follows: grade 0, 0–3; grade 1, 4–6; grade 2, 7–9; and grade 3, 10–12. All scoring was conducted by examining at least ten microscopic fields in each IHC antibody immunostaining. Results of the IHC staining were stratified into groups as follows: FGFR2-low for grades 0–2, high for grades 3 or 4; VEGF-low for grade 0–2 and high for grades 3 or 4; and TRAIL-R1- and TRAIL-R2–low for grades 0–2 and high for grades 3 or 4 (Fig 1).

### Histological assessment of tumor and surrounding nontumor tissue samples

Tumors were evaluated by assessing tumor grade, vessel invasion, number of lesions, and tumor size. Tumor grading followed the Edmonson-Steiner (E-S) grading system. Vascular invasion was defined according to the presence of micro- or macroscopic evidence of blood-vessel intrusion. Tumor size and number of lesions were assessed by macroscopic pathologic investigation of resected livers. Matched nontumor tissues were described in terms of underlying liver disease by grade of fibrosis/cirrhosis, portal inflammation, piecemeal necrosis, and steatosis. Histological evaluation of tumors or surrounding nontumor tissue was conducted on slides stained with hematoxylin and eosin and evaluated by a senior pathologist who was blinded to tissue annotations and prognostic data. Liver fibrosis, portal inflammation, and piecemeal necrosis were assessed according to the Ishak score for chronic hepatitis [25]. Steatosis and lobular infiltration were evaluated according to nonalcoholic fatty liver disease activity score and staging system [26].

### Statistical analysis

All statistical analyses were conducted using SPSS software (version 17, Chicago, IL, SPSS Inc.). Exact χ^2^ testing or Fisher’s exact test was used for categorial data. Observed OS and disease-free survival were estimated with the Kaplan-Meier method and tested with log-rank analysis. Disease-free survival was then investigated using a Cox proportional-hazards regression model. Hazard ratios (HR) and 95% confidence intervals (CI) of these four Cox proportional-hazards regressions are presented. A *p-*value of less than 0.05 was considered statistically significant.

## Results

### Patient characteristics

Demographic and clinicopathological features of the patients are listed in Table 1. The median follow-up period was 47.0 months. Mean age at diagnosis was 57.0 years (range, 30–74). Forty-six (85.2%) patients were positive for hepatitis B virus (HBV) surface antigen, and four were positive for the hepatitis C virus (HCV) antibody. Liver cirrhosis was diagnosed histologically in 38 (70.4%) of the patients. Forty-four (81.4%) and 10 (18.6%) of the patients had Child-Pugh scores of 5 and 6, respectively when the initial hepatic resection was performed. In vascular invasion, only microvascular invasion occurred.

**Table 1:** Clinical characteristics of patients.

		Resected patients (n = 54)	
		n	%
Age at diagnosis	< 60 years	31	57.4
	≥ 60 years	23	42.6
Sex	Male	45	83.3
	Female	9	16.7
Etiology	HBV	46	85.2
	HCV	4	7.4
	Alcohol	2	3.7
	Cryptogenic	2	3.7
Cirrhosis	Yes	38	70.4
	No	16	29.6
Child-Pugh score	5	44	81.4
	6	10	18.6
Modified UICC	Stage I	9	16.7
	Stage II	35	64.8
	Stage III	10	18.5
Tumor size	≤3cm	26	48.1
	3-5cm	13	24.1
	>5cm	15	27.8
Edmonson-Steiner grade	1	6	11.1
	2	31	57.4
	3	15	27.8
	4	2	3.7
Vascular invasion	Yes	23	42.6
	No	31	57.4
Capsule	Yes	32	59.3
	No	22	40.7
Capsule invasion	Yes	17	31.5
	No	37	68.5
Fatty change	Yes	13	24.1
	No	41	75.9
Piecemeal necrosis	Yes	18	33.3
	No	36	66.7
FGFR grade*	Low (0–2)	10	18.5
	High (3–4)	44	81.5
VEGF grade*	Low (0)	24	44.4
	High (1–3)	30	55.6
TRAIL Receptor 1 grade	Low (0–2)	17	31.5
	High (3–4)	37	68.5
TRAIL Receptor 2 grade	Low (0–2)	22	40.7
	High (3–4)	32	59.3
AFP (ng/dL)	< 20	37	68.5
	≥ 20	17	31.5

*IRS (immunoreactive score) = SI (staining intensity) x PP (% of positive cells)

G0, IRS 0–3; G1, IRS 4–6; G2, IRS 7–9; G3, IRS 10–12

### HCC recurrence after liver resection

**Fig 2:**
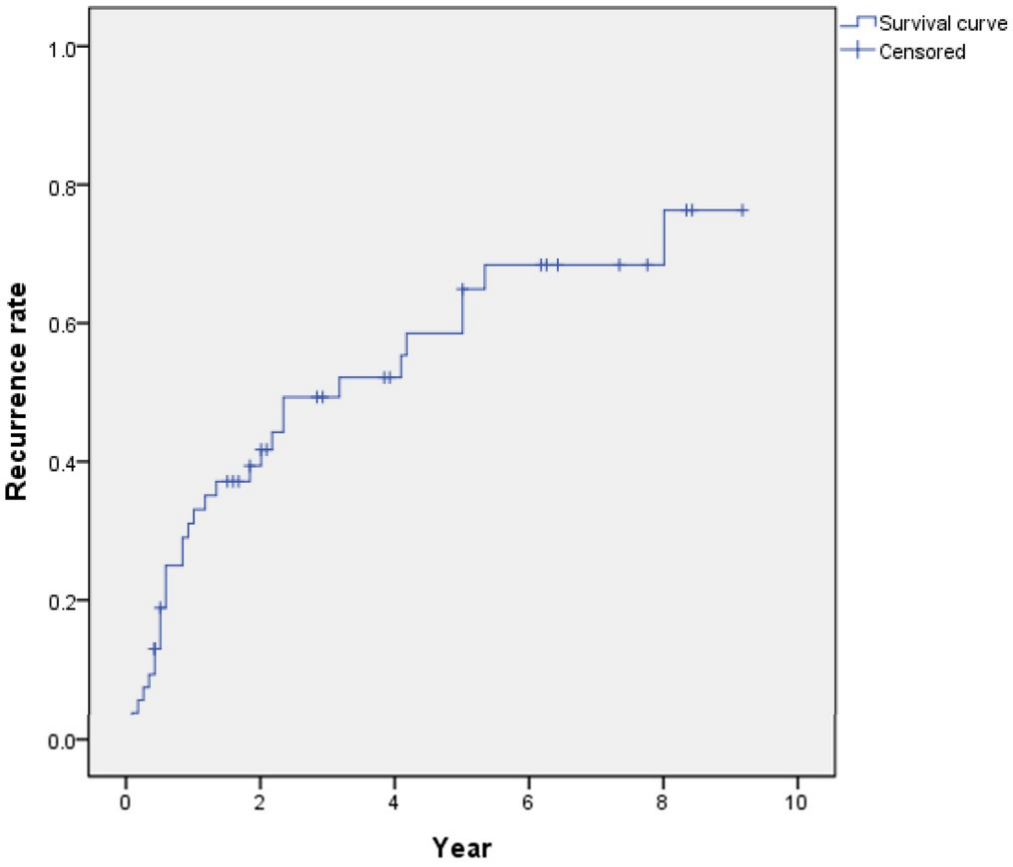
Overall cumulative recurrence rate curve of the 54 HCC patients. Recurrence rates at 1, 2, 3, 4, and 5 years were 32.5, 40.9, 51.0, 53.7, and 66.1%, respectively.

**Fig 3:**
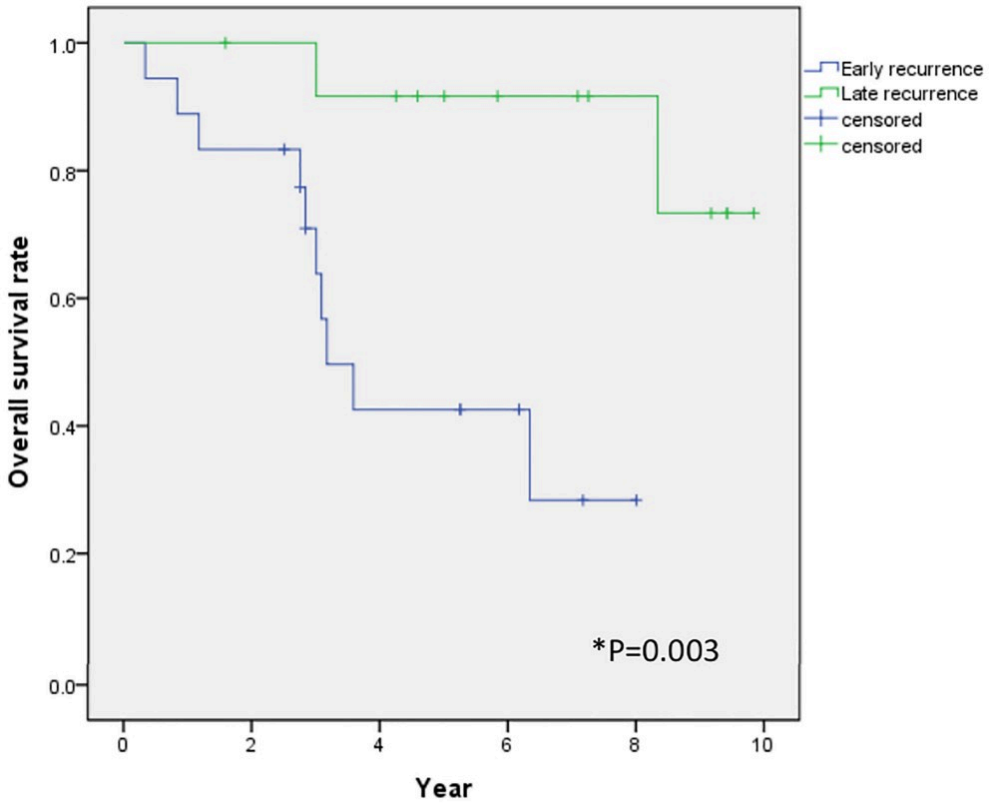
Comparison of overall survival rates between early and late recurrence group. Overall survival in the late recurrence group had better survival than that of early recurrence group (5-year OS: 73.8% vs 28.5%, P = 0.003).

HCC recurrence was observed in 31 (57.4%) patients during the follow-up period, and in all cases, the liver was the first site of recurrence. The overall cumulative recurrence rate curves for all patients are shown in Fig 2. The cumulative 1-, 3-, and 5-year recurrence rates were 32.9%, 53.6%, and 68.1%, respectively. Early recurrence (≤2 year) after HCC resection was observed in 18 of 31 total recurrence patients and late recurrence (>2 year) was observed 13 patients. OS in the late recurrence group had better survival than that of early recurrence group (5-year OS: 73.8% vs 28.5%, P = 0.003) (Fig 3).

### Clinicopathological factors contributing to HCC recurrence after liver resection

**Fig 4:**
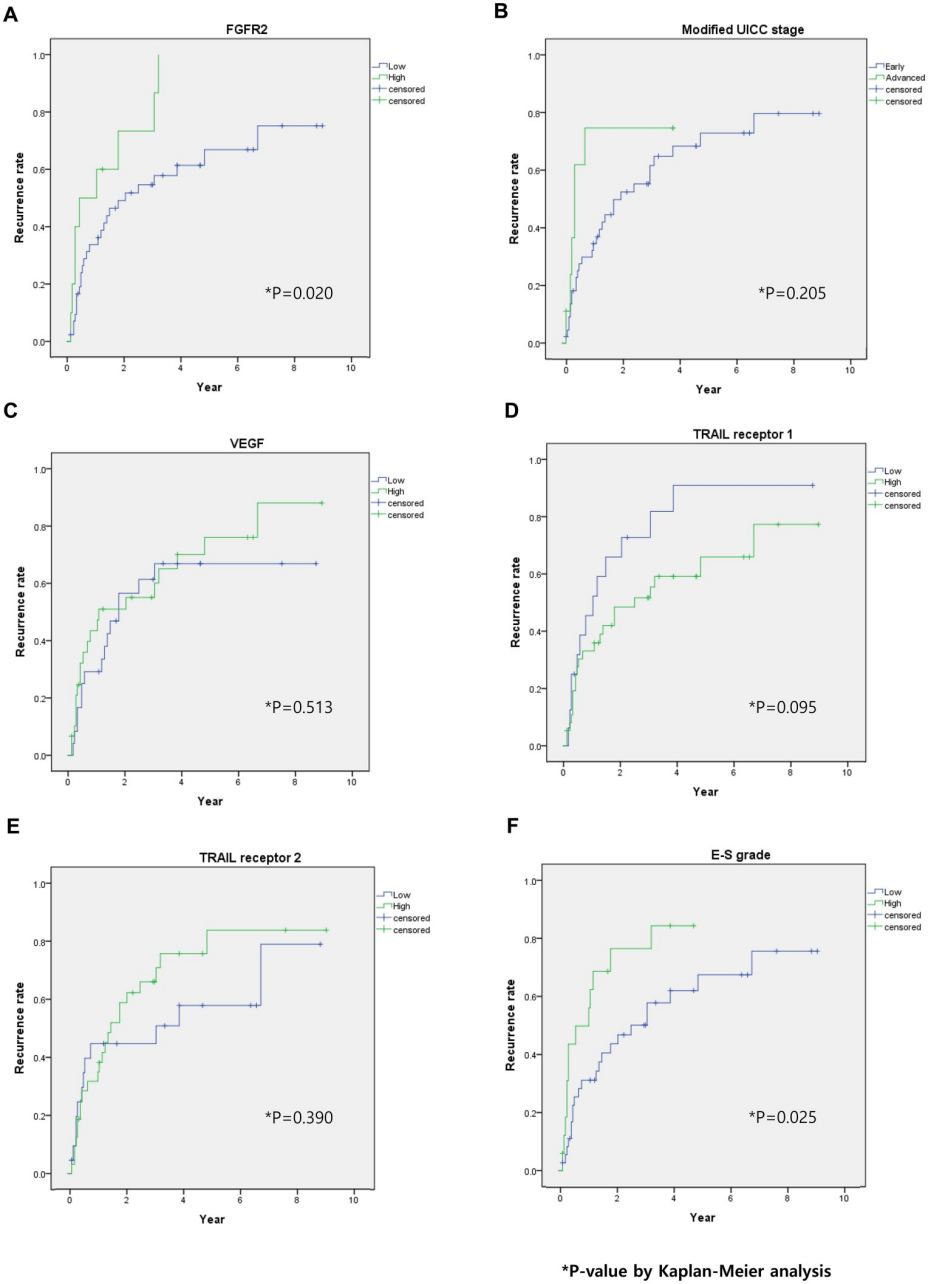
Recurrence rates according to FGFR2 grade, modified UICC, VEGF, TRAIL receptor 1, TRAIL receptor 2, and E-S grade. Kaplan-Meier estimates of (A) FGFR2, (B) modified UICC, (C) VEGF, (D) TARIL-R1, (E) TRAIL-R2, and (F) E-S grade designated as low (or early) or high (or advanced). (A) Increased FGFR2 expression was associated with recurrence. There were no significant differences in recurrence rates related to (B) modified UICC, (C) VEGF, and (E) TRAIL-R2. There was tendency toward high recurrence rate related to (D) low TRAIL-R1, difference not statistically significant. *P*-values were obtained from the log-rank test.

The investigated variables were sex, cirrhosis, AFP, modified UICC stage, E-S grade, vascular invasion, and expression levels of FGFR2, VEGF, TRAIL-R1, and TRAIL-R2. Univariate analyses revealed that FGFR2 expression and E-S grade were the only statistically significant predictive factors for HCC recurrence. Multivariate analysis revealed that a high recurrence rate after hepatic resection was solely correlated with high FGFR2 expression (IRS 3 or 4) (Fig 4A; Table 2). Modified UICC and expression levels of VEGF and TRAIL-R2 were not correlated with recurrence (Fig 4B, 4C and 4E). Although there was a tendency towards a correlation between low TRAIL-R1 (grades 0–2) and HCC recurrence, that correlation did not reach statistical significance (Fig 4D).

**Table 2:** Risk factors for tumor recurrence.

Variable	Univariate analysis			Multivariate analysis		
	HR	95% CI	*P-*value*	HR	95% CI	*P-*value*
Vascular invasion	1.440	0.741–2.800	0.282			
AFP(< 20)	0.890	0.426–1.859	0.757			
E-S grade (> II)	2.175	1.076–4.397	0.030	1.717	0.800–3.687	0.165
Sex	1.138	0.468–2.768	0.776			
Cirrhosis	1.191	0.580–2.446	0.634			
Advanced Modified UICC (> II)	1.756	0.720–4.284	0.216	2.178	0.855–5.552	0.103
High FGFR2	2.411	1.112–5.230	0.026	2.596	1.185–5.687	0.017
High VEGF	1.248	0.637–2.446	0.518			
High TRAIL receptor 1	0.522	0.263–1.034	0.062	0.522	0.259–1.051	0.069
High TRAIL receptor 2	1.357	0.670–2.747	0.396			

* Cox proportional hazards regression analysis

HR, hazard ratio; CI, confidence interval; FGFR2, fibroblast growth factor receptor 2; high FGFR2, grade 3–4; modified UICC, modified Union for International Cancer Control; advanced stage, stage III; high E-S grade, Edmonson-Steiner grade III-IV.

### Correlation between FGFR2 expression and clinicopathological factors in hepatocellular carcinoma patients

Clinicopathological differences between the high and low FGFR2 expression groups were compared (Table 3), and no significant differences were observed for age, sex, modified UICC, capsule invasion, vascular invasion, VEGF grade, TRAIL-R1 grade, TRAIL-R2 grade, or AFP between these two groups. However, increased FGFR2 expression had a marginal association with higher E-S grade (> II) (60% [6/10] vs. 25% [11/44], *p* = 0.056).

**Table 3:** Correlations between FGFR2 expression and clinicopathological factors in HCC patients.

		FGFR2 expression in HCC				
		High expression (n = 10)		Low expression (n = 44)		*p*-value
		n	%	N	%	
Age at diagnosis	< 60 years	8	30.0	24	54.5	0.489
	≥ 60 years	2	70.0	20	45.5	
Sex	Male	8	80.0	37	84.1	0.667
	Female	2	20.0	7	15.9	
Etiology	HBV	10	100.0	36	81.8	0.198
	HCV	0	0	4	9.1	
	Alcohol	0	0	2	4.5	
	Cryptogenic	0	0	2	4.5	
Cirrhosis	Yes	9	90.0	29	65.9	0.249
	No	1	10.0	15	34.1	
Child-Pugh score	5	9	90.0	36	81.8	1.000
	6	1	10.0	8	18.2	
Modified UICC	Stage I	2	20.0	7	15.9	0.488
	Stage II	7	70.0	28	63.6	
	Stage III	1	10.0	9	20.5	
Edmonson-Steiner grade	≤ II	4	40.0	33	75.0	0.056
	> II	6	60.0	11	25.0	
Vascular invasion	Yes	7	70.0	16	36.4	0.078
	No	3	30.0	28	63.6	
Capsule	Yes	5	50.0	27	61.4	0.723
	No	5	50.0	17	38.6	
Capsule invasion	Yes	3	30.0	14	31.8	1.000
	No	7	70.0	30	68.2	
Fatty change	Yes	3	30.0	10	22.7	0.689
	No	7	70.0	34	77.3	
Piecemeal necrosis	Yes	5	50.0	13	29.5	0.215
	No	5	50.0	31	72.2	
VEGF grade*	Low (0)	2	20.0	22	50.0	0.157
	High (1–3)	8	80.0	22	50.0	
TRAIL Receptor 1 grade	Low (0–2)	5	40.0	13	29.5	0.459
	High (3–4)	6	60.0	31	70.5	
TRAIL Receptor 2 grade	Low (0–2)	4	40.0	18	40.9	1.000
	High (3–4)	6	60.0	26	59.1	
AFP (ng/dL)	< 20	5	50.0	32	72.7	0.257
	≥ 20	5	50.0	12	27.3	

*IRS (immunoreactive score) = SI (staining intensity) x PP (% of positive cells)

G0, IRS 0–3; G1, IRS 4–6; G2, IRS 7–9; G3, IRS 10–12

### Clinicopathologic factors contributing to overall survival after liver resection

**Fig 5:**
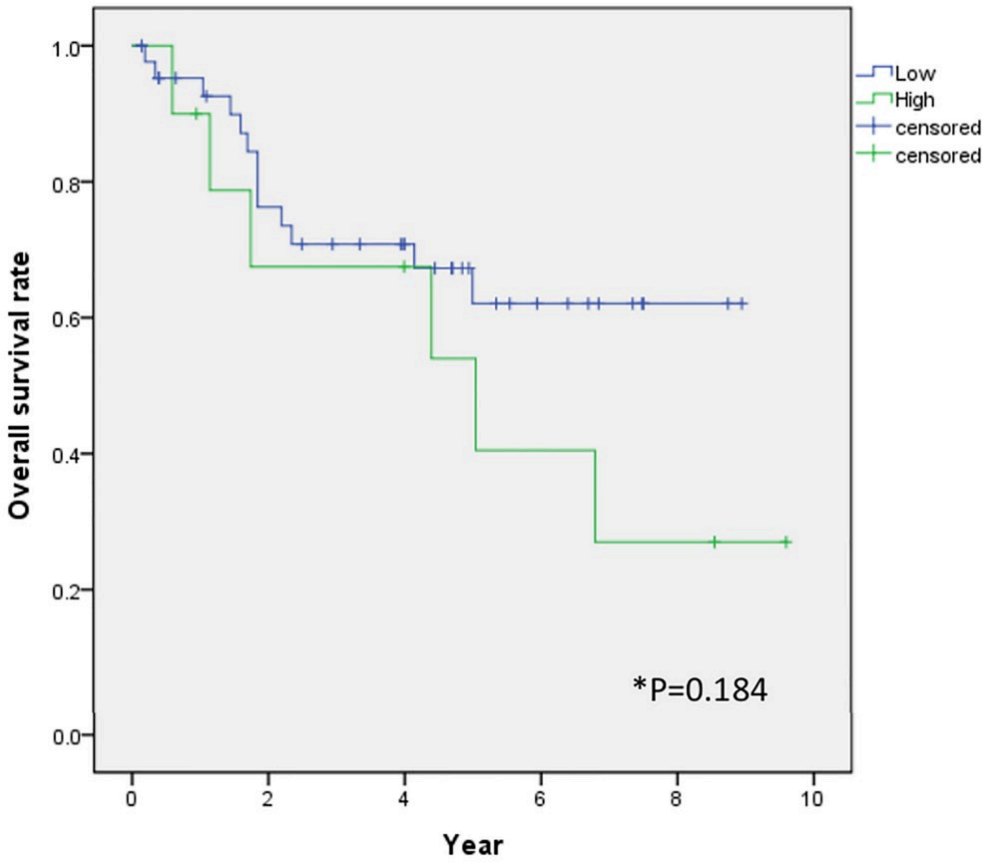
Comparison of overall survival rates between low and high FGFR2 expression levels. Overall survival was not different between low and high FGFR2 expression levels (p = 0.184).

Univariate and multivariate analyses revealed that OS after hepatic resection was correlated with advanced stage as determined by modified UICC classification (> II). High levels of FGFR expression did not affect OS (*p* = 0.242) (Fig 5; Table 4).

**Table 4:** Cox proportional hazards analysis of clinicopathological factors for overall survival.

Variable	Univariate analysis			Multivariate analysis		
	HR	95% CI	*P-*value*	HR	95% CI	*P-*value*
Vascular invasion	1.773	0.614–5.122	0.290			
AFP(>20)	2.464	0.685–8.864	0.167	1.361	0.339–5.459	0.664
Age	1.073	1.006–1.144	0.032	1.048	0.980–1.212	0.174
Edmonson Steiner grade (> II)	1.776	0.615–5.129	0.289			
Sex (Male)	1.252	0.279–5.612	0.769			
Cirrhosis	2.017	0.561–7.247	0.282			
Advanced Modified UICC (>II)	5.552	1.766–17.449	0.003	5.552	1.766–17.449	0.003
High FGFR2	2.025	0.621–6.603	0.242			
High VEGF	2.202	0.634–6.457	0.234			
High TRAIL receptor 1	1.286	0.356–4.649	0.702			
High TRAIL receptor 2	0.724	0.249–2.101	0.552			

* Cox proportional hazards regression analysis

HR. Hazards Ratio; CI, confidence interval; FGFR2, fibroblast growth factor receptor 2; high FGFR2, grade 3–4; modified UICC, modified Union for International Cancer Control; advanced stage, stage III. High E-S grade; Edmonson-Steiner grade, grade III, IV.

## Discussion

Results of this study suggest that expression levels of FGFR2 in post-liver-resection tumor samples are closely related to tumor recurrence; specifically, increased expression of FGFR2 was associated with higher levels of recurrence. Expression levels of other biomarkers, however, such as VEGF, TRAIL-R1, and TRAIL-R2 were not associated with recurrence after hepatic resection. This study also indicated that high FGFR2 expression had a marginal association with more advanced E-S grade (p = 0.056).

The period of recurrence after resection is considered to be important factor. Previous study suggested that early recurrence was associated with intrahepatic metastasis and late recurrence was associated with de novo primary HCC [27, 28]. In this study, we showed that early recurrence group had worse OS than late recurrence group (P = 0.003). This is similar to the result of previous studies. In a study of japan, recurrence period was prognostic factor of OS [28]. Combined with our findings, these data suggest that early recurrence might be associated with intrahepatic metastasis correlated with poor prognosis.

Vascular invasion, both micro- and macroscopic, is considered the strongest predictor of HCC recurrence, although other variables, such as tumor size, number of nodules, AFP level, degree of differentiation, and satellite nodules have also been associated with recurrence [5, 21]. Unfortunately, microvascular invasion and satellite nodules can be assessed only with full pathological specimens, reducing the likelihood of an accurate prediction of HCC recurrence with these variables. More than 80% of patients with HCC presented with an additional life-threatening condition like cirrhosis, confounding prognosis prediction. Certain clinical-based staging systems, in particular, the widely accepted BCLC algorithm [29], address both conditions in establishing a clinical treatment plan. Accurate prognosis prediction is crucial in modern oncology, but data regarding the effects of biomarkers, such as cytokine receptors, signaling proteins, and protein receptors on HCC recurrence in patients with complex solid neoplasms remains scarce.

The present study investigated the prognostic biomarkers FGFR2, VEGF, TRAIL-R1, and TRAIL-R2 alongside clinicopathological data to improve outcome prediction in patients with resected HCC. FGFR2, VEGF, TRAIL-R1, and TRAIL-R2 are known to be associated with carcinogenesis, and some of these tumor tissue biomarkers did help refine recurrence prediction in individuals undergoing surgical resection for HCC.

Our data indicated that FGFR2 expression level could be a predictive factor for recurrence in HCC patients. FGFR2 is active in hepatocytes during liver development and liver regeneration [17], and severe impairments in liver regeneration have been observed in FGFR2 dominant negative transgenic mice [30]. In normal livers of adult rats, FGFR2 mRNA was either undetectable or present at very low levels [31]. Asada et al. demonstrated that FGFR2 was expressed in hepatoma-derived cell lines while healthy human primary hepatocytes did not express FGFR2. His group suggested that HCC proliferation might be regulated by an autocrine or paracrine mechanism mediated by FGF/FGFR2 [32]. In the present study, high levels of FGFR2 expression were associated with poor E-S grade; these findings recapitulate results from a previous study demonstrating that increased FGFR2 expression was significantly correlated with poor histological differentiation, increased incidence of portal-vein invasion, and high levels of AFP [17]. Taken together, these findings indicate that high levels of FGFR2 expression, already associated with poor pathologic differentiation, might increase the likelihood of HCC recurrence.

VEGF participates in tumor proliferation, invasion, and metastasis [33]. TRAIL is a mediator of apoptotic signaling, and Kriegl et al. demonstrated that loss of TRAIL receptors was an independent predictor of survival in patients with HCC undergoing partial hepatectomy [34]. We found previously that HCV antibodies triggered TRAIL-receptor-dependent apoptosis, and that apoptosis was mitigated by TRAIL-receptor siRNA [35]. These findings indicate that TRAIL receptors might be closely related to HCV-induced hepatocarcinogenesis. However, in this study, HCC recurrence was not significantly associated with VEGF, TRAIL-R1, or TRAIL-R2. These results might help improve our understanding of how the biomarkers FGFR2, VEGF, TRAIL-R1, and TRAIL-R2 relate to recurrence, potentially yielding refinements to clinical staging systems and tumor recurrence predictions through improved stratification of HCC patients having undergone hepatic resection.

This study did not find an association between FGFR2 and OS; specifically, no difference in OS rates was observed between high and low expression levels of FGFR2 (*p* = 0.184; Fig 5). However, Harimoto et al. previously reported that OS was better in the low-FGFR2 group than in the high-FGFR2 group [17]. This discrepancy might be due to the fact that both studies only included a small number of patients. We thereby recommend large-scale studies to determine if FGFR2 expression is associated with OS.

In conclusion, FGFR2 expression was found to be an independent predictive factor for HCC recurrence. Furthermore, among the biomarkers FGFR2, VEGF, and TRAIL-R1 and TRAIL-R2 investigated in the present study, FGFR2 was the only one that predicted HCC recurrence. Results also showed that high levels of FGFR2 expression were related to advanced E-S grade. These data indicate that more intensive surveillance for HCC recurrence is warranted in patients with high levels of FGFR2 expression.

## Supporting information

S1 File Raw data. (XLSX)

## Abbreviations

AFP: alpha fetoprotein
BCLC: Barcelona Clinic for Liver Cancer
E-S: Edmonson-Steiner
FGFR2: fibroblast growth factor receptor 2
HCC: hepatocellular carcinoma
IHC: immunohistochemical
TRAIL: tumor necrosis factor related apoptosis-inducing ligand
UICC: Union for International Cancer Control
VEGF: vascular endothelial growth factor
